# Cortical Excitability Dynamics During Fear Processing

**DOI:** 10.3389/fnins.2019.00568

**Published:** 2019-06-04

**Authors:** Venkata C. Chirumamilla, Gabriel Gonzalez-Escamilla, Nabin Koirala, Tamara Bonertz, Sarah von Grotthus, Muthuraman Muthuraman, Sergiu Groppa

**Affiliations:** Section of Movement Disorders and Neurostimulation, Biomedical Statistics and Multimodal Signal Processing Unit, Department of Neurology, Focus Program Translational Neurosciences, University Medical Center, Johannes Gutenberg University Mainz, Mainz, Germany

**Keywords:** TMS, EEG, instructed fear paradigm, resilience, functional connectivity

## Abstract

**Background:**

Little is known about the modulation of cortical excitability in the prefrontal cortex during fear processing in humans. Here, we aimed to transiently modulate and test the cortical excitability during fear processing using transcranial magnetic stimulation (TMS) and brain oscillations in theta and alpha frequency bands with electroencephalography (EEG).

**Methods:**

We conducted two separate experiments (no-TMS and TMS). In the no-TMS experiment, EEG recordings were performed during the instructed fear paradigm in which a visual cue (CS+) was paired with an aversive unconditioned stimulus (electric shock), while the other visual cue was unpaired (CS-). In the TMS experiment, in addition the TMS was applied on the right dorsomedial prefrontal cortex (dmPFC). The participants also underwent structural MRI (magnetic resonance imaging) scanning and were assigned pseudo-randomly to both experiments, such that age and gender were matched. The cortical excitability was evaluated by time-frequency analysis and functional connectivity with weighted phase lag index (WPLI). We further linked the excitability patterns with markers of stress coping capability.

**Results:**

After visual cue onset, we found increased theta power in the frontal lobe and decreased alpha power in the occipital lobe during CS+ relative to CS- trials. TMS of dmPFC increased theta power in the frontal lobe and reduced alpha power in the occipital lobe during CS+. The TMS pulse increased the information flow from the sensorimotor region to the prefrontal and occipital regions in the theta and alpha bands, respectively during CS+ compared to CS-. Pre-stimulation frontal theta power (0.75–1 s) predicted the magnitude of frontal theta power changes after stimulation (1–1.25 s). Finally, the increased frontal theta power during CS+ compared to CS- was positively correlated with stress coping behavior.

**Conclusion:**

Our results show that TMS over dmPFC transiently modulated the regional cortical excitability and the fronto-occipital information flows during fear processing, while the pre-stimulation frontal theta power determined the strength of achieved effects. The frontal theta power may serve as a biomarker for fear processing and stress-coping responses in individuals and could be clinically tested in mental disorders.

## Introduction

Fear is an emotional response that is triggered in the brain in anticipation of a potentially dangerous event (Garcia, 2017). Instructed fear paradigms are commonly the experimental choice to study the adaptive capacity of human brain processing during threat. In such paradigms, the participants are explicitly informed that a conditioned stimulus (CS+) will be repeatedly paired with an aversive unconditioned stimulus (US), while a second conditioned stimulus will always be safe (CS-) (Mechias et al., 2010; Mertens et al., 2018). These fear responses are well associated with subjective and peripheral psycho-physiological measures, in terms of skin conductance, heart rate acceleration and self-reported fear ratings (Gonzalez-Escamilla et al., 2018a).

Accumulating evidence indicates that instructions about the CS+/US contingency evoke effects on the neural activity of a distributed network of brain regions, namely the amygdala, the cingulate cortex, the insula, hippocampus and prefrontal cortices, among which the dorsomedial prefrontal cortex (dmPFC) plays an important role in fear processing by dynamically regulating excitability (Phelps et al., 2001; Gonzalez-Escamilla et al., 2018b). A recent electroencephalography (EEG) study showed that increased theta power in frontal regions together with decreased alpha power at occipital locations are potential attributes of instructed fear responses in humans (Chien et al., 2017). However, the modulation of the neural oscillations during fear processing remains unclear. Furthermore, the individual stress coping abilities as measured by the brief resilience scale (BRS) are negatively correlated with anxiety and depression (Chmitorz et al., 2018), suggesting that this measure may be useful in searching for behavioral markers of brain circuit responses during potential threatening events. Transcranial magnetic stimulation (TMS) is a non-invasive stimulation technique that offers targeted modulation of cortical brain regions in humans (Bergmann et al., 2016). The combined TMS-EEG technique can be used to track the cortical excitability and functional connectivity alterations of the stimulated brain region (Groppa et al., 2013; Pellicciari et al., 2017).

In a previous study, we analyzed the TMS-evoked potentials (TEP) measured with EEG and TMS over the right dmPFC during an instructed fear paradigm (Gonzalez-Escamilla et al., 2018a). We showed that TMS over dmPFC led to increased evoked cortical excitability at a specific time window during CS+ relative to CS-, measured by TMS-EEG potentials amplitudes and latencies. Moreover, the enhanced responses were further supported by the underlying structural integrity of the brain. On the basis of these results, in the current study we focused on characterizing the transient modulated oscillatory activity and functional connectivity alterations following single-pulse TMS during an instructed fear paradigm. First, we performed an instructed fear paradigm together with EEG (no-TMS experiment) to determine the optimal time window for modulatory effects of TMS in a subsequent experiment. Based on evidence from the no-TMS experiment, we adapted the instructed fear paradigm together with the application of TMS over the right dmPFC in a second group of participants (TMS experiment) to determine the causal alterations during the fear conditioning task. We then analyzed pre-stimulation frontal theta power to predict the oscillatory activity in the frontal cortex to show that the brain response to TMS is state dependent. Moreover, we correlated individual transients of modulated oscillatory activity to BRS ratings to reveal the relationship between frontal theta power and individual stress coping abilities.

## Subjects and Methods

### Subjects

Thirty-eight healthy subjects (no-TMS experiment: *n* = 19, 9 males, mean age 27.4 ± 4.32 years, TMS experiment: *n* = 19, 10 males, mean age 28.6 ± 6.64 years) were included in our study. All participants had two visits to the lab. During the first visit, MRI data was acquired, whereas, at the second visit, an instructed fear paradigm (no-TMS experiment) or an instructed fear paradigm together with TMS (TMS experiment) was performed. Participants were assigned to one of the two experiments pseudo-randomly, such that age and gender were matched. The TMS experiment was conducted after completing the no-TMS experiment.

### MRI Data Acquisition

Magnetic resonance images were acquired for the purpose of neuronavigation using a 3 Tesla MRI scanner (Magnetom Tim Trio, Siemens Healthcare, Erlangen, Germany) equipped with a 32-channel head coil at the Neuroimaging Center (NIC) in Mainz, Germany. A magnetization-prepared rapid gradient-echo (MP-RAGE) sequence was used (repetition time [TR] = 1900 ms; echo time [TE] = 2.54 ms; inversion time [IT] = 900 ms; pixel bandwidth = 180; acquisition matrix = 320 × 320; flip angle = 9°; pixel spacing = 0.8125 × 0.8125 mm; slice thickness = 0.8 mm).

### Experimental Procedure (Instructed Fear Paradigm)

**FIGURE 1:**
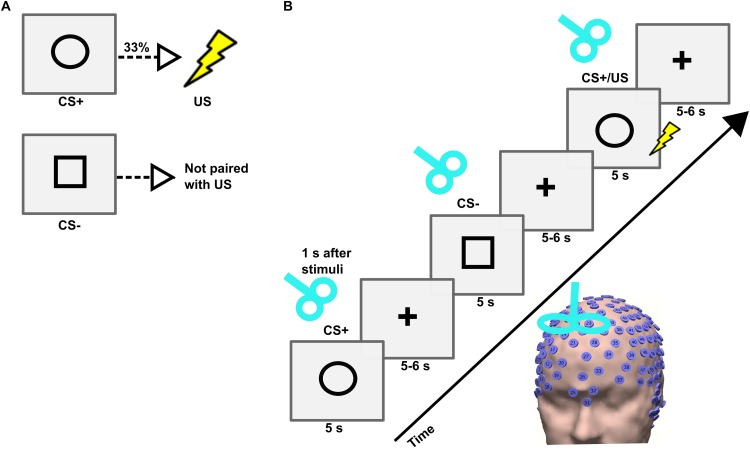
**(A)** Conditioned stimulus (CS+), unconditioned stimulus (US), and neutral stimulus (CS–) and their contingencies in an instructed fear paradigm. **(B)** Trial sequence in the TMS experiment. Each trial consisted of the presentation of a stimulus (CS+ or CS–) followed by a fixation cross. The stimulus was presented on a computer screen for 5 s followed by a fixation cross that jittered between 5 and 6 s. A single-pulse TMS was applied on the right dorsomedial prefrontal cortex (dmPFC) 1 s after the onset of each stimulus.

The instructed fear paradigm was developed with the Cogent toolbox^1^ in Matlab 2006b (MathWorks). First, the participant was asked to sit on a chair, and an electric shock was applied to the dorsal part of the left hand using a surface electrode that was connected to a DS7A electrical stimulator (Digitimer). Then, the participant was requested to rate the amount of pain perceived on a scale from 0 (no pain) to 10 (intense pain). An electric shock intensity corresponding to a pain level of 7 was employed during the experiment (Meyer et al., 2015). The experiment consisted of two visual cues, namely a circle and a square, presented in a pseudo-randomized order on a screen for 5 s with an inter-trial interval (ITI) jittered between 9 and 11 s (Figure 1A,B). Before the beginning of the experiment, all participants were instructed that a CS+ (visual cue circle) would be associated with a US (electric shock) with a probability of 33% during the time where the visual cue was present on the screen; and that the CS- (visual cue square) would never be associated with a shock. The visual cues were counterbalanced across subjects. A total of 90 visual cues (54 CS+ including 18 CS+/US, and 36 CS-) were used. During the whole duration of the experiment EEG signals were recorded with a high-density (256 electrodes) EEG system (Net Station 5.0, EGI, United States). The caps were placed manually with the Cz electrode positioned over a centralized location on the scalp, which was determined as the simultaneous midpoint of the arc length for both nasion-inion and preauricular arcs. The electrode impedances were kept under 50 KΩ throughout the experiment (Ferree et al., 2001), and a sampling frequency of 250 Hz was applied. The time of experiment across subjects was uniformly distributed throughout the day between morning and evening.

### Single-Pulse TMS

In the TMS experiment, single-pulse TMS was applied over the right dmPFC after 1 s from each visual cue onset. The TMS was delivered using a Magstim Super Rapid^2^ stimulator (Magstim, United Kingdom) through a figure-of-eight coil with internal wing diameter of 70 mm. First, the resting motor threshold (RMT) was determined as the minimum stimulus intensity required to elicit motor evoked potentials of amplitude 50 μV in 5 out of 10 consecutive trials at rest (Groppa et al., 2012). The TMS was targeted on the right dmPFC (MNI coordinate: *x* = 10, *y* = 12, *z* = 58) (Meyer et al., 2018). We used TMS-Navigator (Localite, Sankt Augustin, Germany) based on a coregistered individual T1-weighted MRI to navigate the TMS coil and to maintain its exact location and orientation throughout an experimental session. The TMS pulses were applied with a stimulation intensity of 110% of RMT. All participants wore ear plugs during the entire TMS experiment to reduce the auditory click sound produced by TMS pulse.

### EEG Data Processing

**FIGURE 2:**
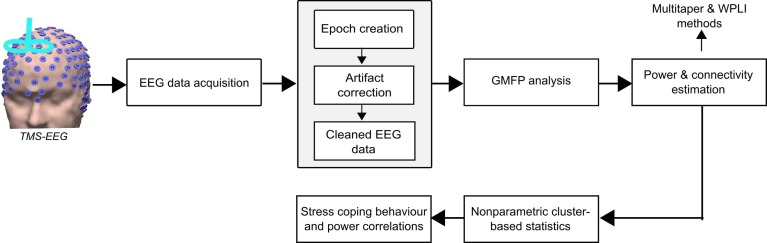
Electroencephalography data were acquired from the no-TMS and TMS experiments. The data were segmented into epochs and excluded the TMS-related artifacts (ringing, decay, and muscle), and non-TMS artifacts (eye-blinks). Afterward, the global mean field power (GMFP) was calculated across all EEG channels for both the experiments. Further, the power and connectivity were assessed by the multitaper method with Hanning window and weighted phase lag index (WPLI), respectively. The significant differences in power and connectivity between the conditions were tested with non-parametric cluster-based statistics. Finally, the neural oscillatory power was correlated with individual stress coping capabilities.

The processing steps of the analysis pipeline performed in this study are shown in Figure 2. Pre-processing of the EEG data was performed using a systematic procedure described elsewhere (Herring et al., 2015). In brief, it included the following steps: epoch creation, exclusion of TMS-related artifacts, and physiological artifacts. The EEG data from both experiments were preprocessed using the Fieldtrip toolbox^2^ and MATLAB R2015b (The MathWorks). The EEG data was cut in epochs of 7 s within the time interval of -2 to 5 s from the onset of the visual cue (Bai et al., 2017). In the TMS experiment, the 0.025 s of EEG signal containing the TMS pulse ringing artifacts were deleted (-0.005 to 0.02 s around TMS pulse onset). In both experiments, the original trials in which the actual US was applied were discarded. Thus, only the condition-specific (CS+ and CS-) trials were considered in further analysis. Then, EEG data were re-referenced to a common grand average of all electrodes. All trials were visually inspected, and the artefactual electrode data were interpolated using the spherical spline interpolation method (Perrin et al., 1989). Independent component analysis (ICA) was performed using the FastICA method and the components reflecting eye-blinks, saccades and decay artifacts (for the TMS experiment) were discarded (Bai et al., 2016). The remaining components were transformed back into electrode data representation. In the TMS experiment, the remaining muscle artifact due to TMS pulse after ICA was removed and interpolated using the pchip (Piecewise Cubic Hermite Interpolating Polynomial) method (Herring et al., 2015). Finally, we also implemented a 50 Hz notch filtering to remove the line noise.

In this study, our main goal was to investigate the global and local cortical excitability dynamics during fear processing. Accordingly, we first computed the global mean field power (GMFP) that is a measure of global cortical excitability. Afterward, we assessed the local cortical excitability specifically in the frontal and occipital lobes by estimating the power in theta and alpha frequency bands, respectively. In addition, we also investigated the direction of information flow in these frequency bands with the weighted phase lag index (WPLI) method. The WPLI works based on phase synchronization and is less sensitive to uncorrelated noise sources and has increased statistical power to detect the alterations in phase compared to other methods, such as phase lag index (PLI) (Vinck et al., 2011). Furthermore, the non-parametric tests were chosen due to the fact that they are able to solve the multiple comparisons problem and are highly sensitive to the expected effect (Maris and Oostenveld, 2007).

### Fear Ratings and Heart Rate Estimation

At the end of the experiment, all the subjects were asked to rate their perceived level of fear during the experiment, for both CS+ and CS- independently, on a scale from 0% (not fearful/safe) to 100% (very fearful). Heart rate was estimated from the EEG signals using the extended version of the ICA algorithm (Phelps and LeDoux, 2005) based on information maximization (Chien et al., 2017). In the case of EEG signals, volume conduction is thought to be linear and instantaneous and the sources of cardiac signals are not time-locked. Because the sources of EEG activity are thought to reflect the activity of cortical neurons (Milad et al., 2006), the ICA algorithm can accurately identify the time courses of activation and the scalp topographies of relatively large and temporally independent sources from simulated scalp recordings, even in the presence of a large number of low-level and temporally independent source activities (Palazzo et al., 2008).

For EEG analysis, the EEG signals recorded at the 256 electrodes represent the rows of the sensor input matrix y for the ICA component extraction, the rows of the output data matrix v = Xy are time courses of activation of the ICA components, and the columns of the inverse matrix, X^-1^, give the projection strengths of the respective components onto the scalp sensors.

In general, and unlike principal component analysis (PCA), the component time courses of activation will be non-orthogonal. Corrected EEG signals can then be derived as y′ = (X)^-1^v′, where v′ is the matrix of activation waveforms, v, with rows representing cardiac artefactual sources which are then extracted for further estimations from each participant. In total for the no-TMS experiment, we concatenated the 36 CS+ trials to have 180 s and 36 CS- trials to have 180 s. For the TMS experiment, we concatenated the 36 CS+ trials to have 180 s and 36 CS- trials to have 180 s.

### Global Mean Field Power (GMFP)

The GMFP is an index of distinctive global cortical excitability and also reflects the synchronous activity across observations in response to a specific stimulus (Romero Lauro et al., 2014; Varoli et al., 2018), and was calculated for the time (-0.25 to 2 s) for both CS+ and CS- conditions separately.

### Time-Frequency Analysis

Dynamic changes in neural oscillatory activity were assessed by analyzing the time-frequency representations (TFR) of power. The time-frequency analysis was performed using a multitaper method (Herring et al., 2015). The EEG data were multiplied with hanning tapered sliding window moving in steps of 0.02 s and the length of the time window changed with frequency (*T* = 3 cycles = 3/f). The TFRs were computed for the time range of -0.25 to 2 s for frequencies from 4 to 13 Hz. The time-frequency grand averages across subjects were then computed for both CS+ and CS- conditions separately. The relative baseline correction was applied by dividing the post-visual cue onset power with pre-visual cue onset power (-0.25 to 0 s). In this study, the theta and alpha oscillations were investigated in the frontal and occipital lobe, respectively. The electrodes corresponding to the frontal and occipital lobes are shown (Supplementary Figure 1).

### Functional Connectivity

The WPLI is a functional connectivity measure and evaluates the distribution of phase angle differences between two signals (Vinck et al., 2011). Specifically, if two signals are uncorrelated, the angular difference will be evenly distributed giving a value of zero, whereas if the signals are strongly coupled, the difference will demonstrate an asymmetric distribution, approaching a value of 1 or -1. For computation of the pairwise sensor connectivity, the significant electrodes obtained by comparing power distributions of CS+ and CS- using non-parametric analysis were used as a reference. Then, the WPLI was computed between the reference electrodes and the remaining EEG electrodes at each the theta and alpha bands in both CS+ and CS- conditions, respectively.

### Brief Resilience Scale

All subjects completed a BRS questionnaire (Park et al., 2018). The BRS consists of six questions and is used to characterize the ability of an individual to recover from stressful events (Smith et al., 2008). The procedure for calculation of individual BRS scores has been described elsewhere (Smith et al., 2008).

### Statistical Analysis

Statistical analysis of the data was performed using Statistica software (version 13.1^3^). *Post hoc* tests were performed if the *F*-values were significant (*p* < 0.05) with the Bonferroni correction method, unless otherwise explicitly specified. To study the differences between the stimulus conditions (CS+ and CS-) in behavioral fear ratings and heart rate, we conducted paired *t*-tests. To examine the global cortical excitability dynamics over time windows during fear processing, a one-way repeated measures analysis of variance (rmANOVA) was conducted with the factor Time (9 levels: -0.25–0, 0–0.25, 0.25–0.5, 0.5–0.75, 0.75–1, 1–1.25, 1.25–1.5, 1.5–1.75, and 1.75–2 s), and a dependent measure of GMFP difference between stimulus conditions. To study the differences between stimulus conditions and also across temporal windows, a two-way rmANOVA was run including two factors, Condition (2 levels: CS+ and CS-), and Time, with the dependent measures for separate variables, power in the theta and in the alpha band at the frontal and occipital lobe electrodes (shown in Supplementary Figure 1), respectively. The factor Experiment (2 levels: no-TMS and TMS) was added as a categorical factor. To provide surface topography, we tested the significant differences between stimulus conditions in power and WPLI, using non-parametric cluster-based statistics with the Monte-Carlo method in theta and alpha frequency bands (Maris and Oostenveld, 2007). We applied 500 random permutations in the Monte Carlo method to correct for multiple comparisons, and a threshold of 2 channels to be considered a cluster. We performed the subsequent regression and correlation analyses on the data averaged across all subjects and significant electrodes identified by cluster-based statistics. We conducted a linear regression analysis to investigate the association of theta power before and after the TMS pulse. The four temporal windows (0–0.25, 0.25–0.5, 0.5–0.75, and 0.75–1 s) were added as predictor variables and the window (1–1.25 s) as a dependent variable for the power difference between stimulus conditions as a dependent measure. To assess the relationship between theta power and resilience, the Pearson correlation coefficient was computed between individual theta power difference between conditions and BRS ratings.

## Results

### Fear Ratings and Heart Rate

**FIGURE 3:**
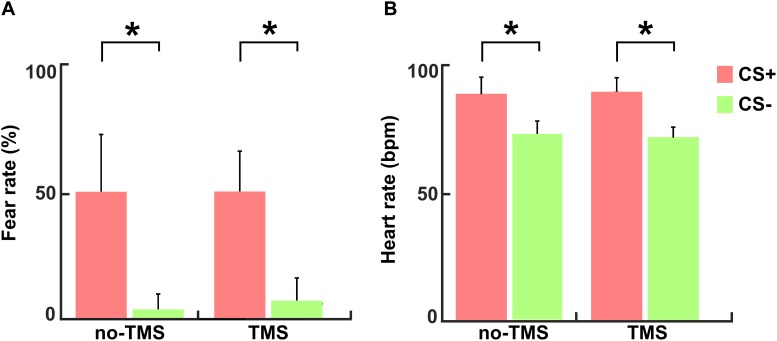
For both experiments (no-TMS and TMS) the **(A)** subjective fear ratings and **(B)** heart rates. The light red bar represents CS+ and light green denotes CS–. The values are mean ± standard deviation. The asterisk (^∗^) denotes significant differences after correcting for multiple comparisons (*p* < 0.001, Bonferroni corrected).

The mean values of subjective fear ratings (+S.D.) for the CS+ and CS- conditions in the no-TMS experiment were 50.2 + 26.9 and 2.6 + 8 and in the TMS experiment 50.5 + 17.5 and 6.8 + 8.8, respectively. The reported fear ratings evidenced well induced fear in the participants during the CS+ condition in comparison to the CS- condition in both experiments (no-TMS experiment: *t*(36) = 17.42; *p* < 0.001; TMS experiment: *t*(36) = 18.47; *p* < 0.001), as shown in Figure 3A. The mean values of the heart rate (+S.D.) in beat per minute (bpm) for the CS+ and CS- conditions in no-TMS experiment were 90 + 6.7 and 74 + 4.7 and in the TMS experiment were 91 + 6.8 and 72 + 4.6, respectively. Accordingly, in both experiments, increased in heart rate was detected during the CS+ trials relative to CS- (no-TMS experiment: *t*(36) = 6.26; *p* < 0.001; TMS experiment: *t*(36) = 5.98; *p* < 0.001) (Figure 3B).

### Global Mean Field Power

**FIGURE 4:**
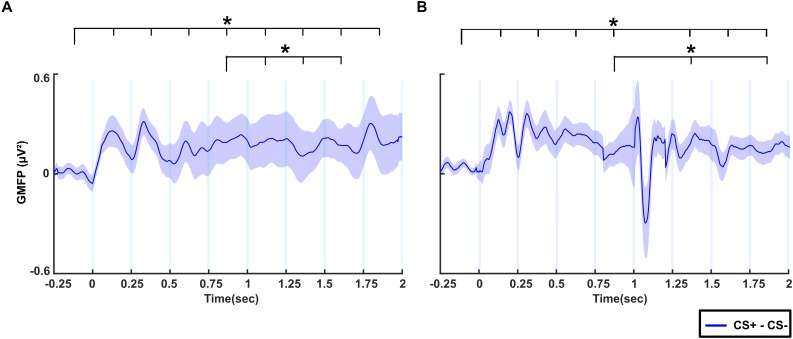
The difference of global mean field power (GMFP) between CS+ and CS– for **(A)** no-TMS experiment and **(B)** TMS experiment. The curve shadings are mean ± standard error of the mean. The asterisk (^∗^) denotes significant differences after correcting for multiple comparisons (*p* < 0.05, Bonferroni corrected).

Figure 4A shows the difference in GMFP between CS+ and CS- conditions across time windows in the no-TMS experiment. The results of the one-way rmANOVA showed a significant main effect of Time (*F*(8, 55) = 4015.7, *p* < 0.001). *Post hoc* testing showed that the difference of GMFP increased in all the time windows (0–2 s) compared to the baseline time window (-0.25–0 s; all at *p* < 0.001). Furthermore, the GMFP difference reduced in time windows (1–1.75 s) relative to (0.75–1 s) window (all at *p* < 0.05). We repeated the same analysis for TMS experiment (Figure 4B). One-way rmANOVA revealed a significant main effect for the factor Time (*F*(8,55) = 7404.1, *p* < 0.001). The *post hoc* comparisons showed that the GMFP difference increased significantly in the time windows (0–0.25, 0.25–0.5, 0.5–0.75, 0.75–1, 1.25–1.5, 1.5–1.75, and 1.75–2 s) compared to the (-0.25–0 s) time window (all at *p* < 0.05). Moreover, the GMFP difference increased significantly in the time windows (1.25–1.5 and 1.75–2 s) compared to the time window (0.75–1 s; all at *p* < 0.05) after single-pulse TMS.

### Neural Oscillations During No-TMS and TMS Experiments

**FIGURE 5:**
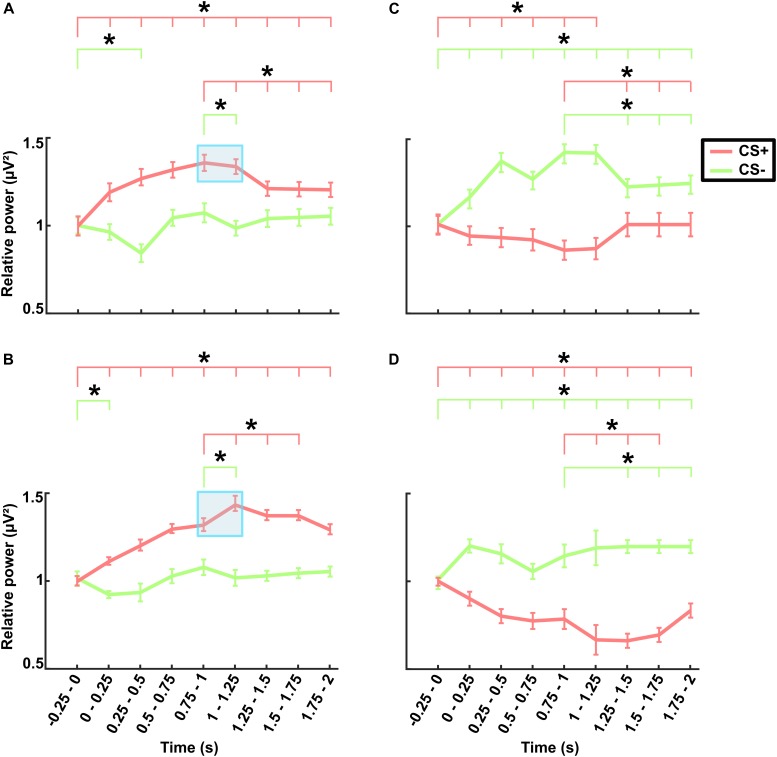
Electroencephalography power averaged across frontal electrodes (shown in Supplementary Figure 1) in the theta band for **(A)** the no-TMS experiment and **(B)** the TMS experiment. The power averaged across occipital electrodes (shown in Supplementary Figure 1) in the alpha band for **(C)** the no-TMS experiment and **(D)** the TMS experiment. The values are mean ± standard error of the mean. The light red bar represents CS+ and light green denotes CS–. The asterisk (^∗^) denotes significant differences after correcting for multiple comparisons (*p* < 0.05, Bonferroni corrected).

The grand averaged theta power for the time (-0.25–2 s) with respect to stimulus onset across frontal lobe electrodes are presented separately for different stimuli (CS+ and CS-) in the no-TMS experiment (Figure 5A) and TMS experiment (Figure 5B). The results of the two-factorial rmANOVA revealed a significant main effect for the stimulus Condition (*F*(1, 18) = 619.7, *p* < 0.001) and a significant main effect for the factor Time (*F*(8, 144) = 240.1, *p* < 0.001). The interaction between the factors was also significant (*F*(8, 144) = 1626.2, *p* < 0.001). The *post hoc* comparisons revealed that in the no-TMS experiment, the theta power was higher during CS+ in all the temporal windows (0–2 s) compared to the baseline time window (-0.25–0 s; all at *p* < 0.05) (Figure 5A). The frontal lobe theta power was significantly lower during CS- in the time window (0.25–0.5 s) compared to the baseline time window (-0.25–0 s; *p* < 0.05). Furthermore, the theta power was reduced during CS+ in the time windows (1–2 s) compared to the (0.75–1 s) time window. And also, the frontal theta power was decreased for CS- in the window (1–1.25 s) compared to the time window (0.75–1 s). In the TMS experiment, frontal theta power was higher during CS+ in all the temporal windows (0–2 s) relative to baseline window (-0.25–0 s), while for CS- it was decreased in the time window (0–0.25 s) compared to baseline window (all at *p* < 0.05) (Figure 5B). As a result of single-pulse TMS, the theta power was higher in frontal lobe during CS+ in the time windows (1–1.75 s) relative to the time window (0.75–1 s) and decreased in the time window (1–1.25 s) during CS- (all at *p* < 0.05).

**FIGURE 6:**
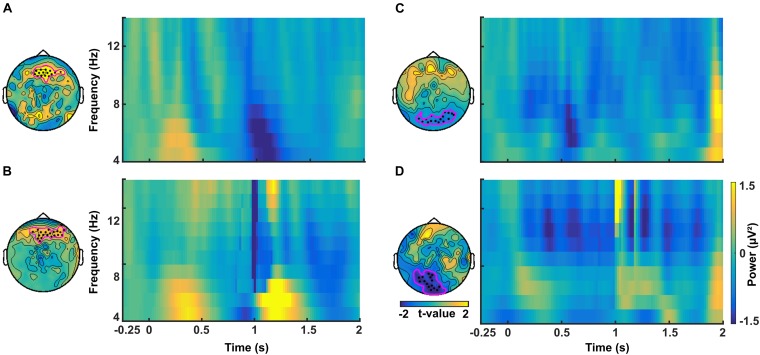
Time-frequency plots of difference in power between CS+ and CS– averaged across channels in the theta band for **(A)** the no-TMS experiment and **(B)** the TMS experiment. In the alpha band, the time-frequency plots of difference in power between CS+ and CS– averaged across channels for **(C)** no-TMS experiment and **(D)** TMS experiment. The considered channels are indicated in each plot. These electrodes were identified with cluster-based statistics (*p* < 0.05).

In a similar manner, we investigated the alpha power dynamics across occipital electrodes in the no-TMS experiment (Figure 5C) and TMS experiment (Figure 5D). Two-way rmANOVA revealed a significant main effect for the factor stimulus Condition (*F*(1, 18) = 4518.2, *p* < 0.001), significant main effect for the factor Time (*F*(8, 144) = 2391.2, *p* < 0.001) and the interaction between factors was also significant (*F*(8, 144) = 2391.2, *p* < 0.001). The *post hoc* tests showed a significant decrease in occipital alpha power during CS+ in the time window (0–1.25 s) but an increase for CS- in the (0–2 s) compared to a baseline time window (-0.25–0 s; all at *p* < 0.05) (Figure 5C). In the time window (1.25–2 s) the occipital alpha power was higher for CS+, and was lower for CS- relative to the (0.75–1 s) time window (all at *p* < 0.05). In the TMS experiment, the occipital alpha power was decreased for CS+ while increased for CS- in all the time windows (0–2 s) compared to a baseline time window (-0.25–0 s; all at *p* < 0.05) (Figure 5D). Due to single-pulse TMS, the occipital alpha power was significantly reduced for CS+ in the time windows (1–1.75 s), while increased for CS- in the time windows (1.25–2 s) compared to the time window (0.75–1 s; all at *p* < 0.05). Further to providing surface topography of the oscillatory power changes, we performed comparisons between stimulus conditions in the theta and alpha frequency bands. In both experiments, statistical comparison of stimulus conditions CS+ and CS- revealed an increase in theta power and a decrease of alpha power in the latency range 0.15–0.45 s (all at *t*_18_ > 2.2, *p* < 0.05) (Figure 6A–D and Table 1).

**Table 1:** Significant clusters (*p* < 0.05) between conditions (CS+ and CS-) that were compared regarding power in theta and alpha frequency bands using cluster-based statistics.

	Cluster latency (s)	Type of cluster	Number of channels in cluster
**Theta**			
no-TMS experiment	0.15–0.45	positive	15
TMS experiment	0.15–0.45	positive	20
**Alpha**			
no-TMS experiment	0.15–0.45	negative	14
TMS experiment	0.15–0.45	negative	15


### Functional Connectivity During No-TMS and TMS Experiments

**FIGURE 7:**
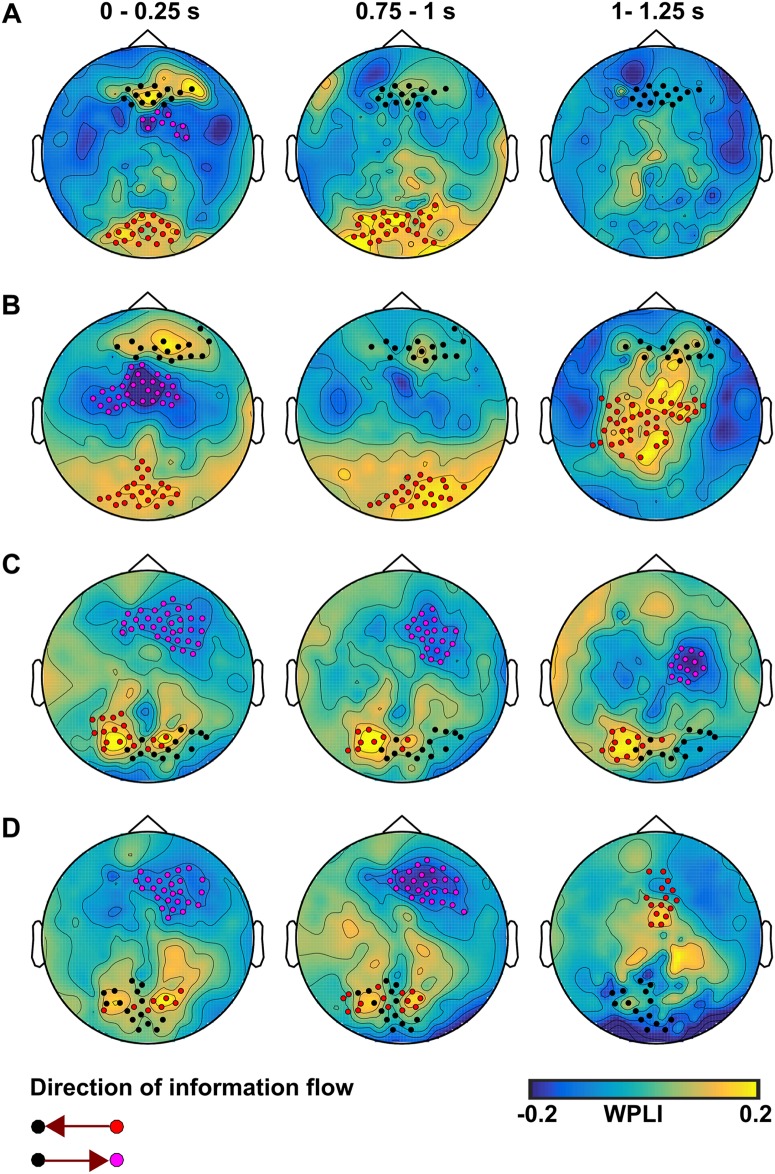
The information flow at EEG electrode level during CS+ compared to CS– in the theta band for **(A)** the no-TMS experiment and **(B)** the TMS experiment. In the alpha band, the information flow is shown for **(C)** the no-TMS experiment and **(D)** the TMS experiment. The time windows are labeled above the plot, while the direction is indicated below. The highlighted electrodes were identified with cluster-based statistics (*p* < 0.05).

The pairwise cluster-based analysis revealed significant differences in dynamic information flow at electrode level between the CS+ and CS- in theta and alpha frequency bands. In both the no-TMS and TMS experiments, we found increased information flow during CS+ relative to CS- from occipital regions to prefrontal regions in the time windows (0–0.25 and 0.75–1 s), while the premotor regions received information from prefrontal regions in the first temporal window (0–0.25 s) after visual cue onset in the theta band (all at *t*_18_ > 2.2, *p* < 0.05) (Figure 7A,B and Table 2). In the same frequency band, the TMS pulse on the right dmPFC increased the information flow from the sensorimotor area and Supplementary Motor Area to the prefrontal regions in the temporal window (1–1.25 s) during CS+ compared to CS- (*t*_18_ > 2.2, *p* < 0.05) (Figure 7B and Table 2). In the alpha band, we found increased information flow form parietal regions to the occipital regions and from there to the prefrontal regions during CS+ relative to CS- in the time windows (0–0.25 and 0.75–1 s) for both the experiments (all at *t*_18_ > 2.2, *p* < 0.05) (Figure 7C,D and Table 2). Moreover, the single-pulse TMS increased the information flow from sensorimotor area to occipital regions in the time window (1–1.25 s) during CS+ compared to CS- in the alpha band (*t*_18_ > 2.2, *p* < 0.05) (Figure 7D and Table 2).

**Table 2:** Significant clusters (*p* < 0.05) between conditions (CS+ and CS-) that were compared regarding weighted phase lag index (WPLI) in theta and alpha frequency bands using cluster-based statistics.

	Cluster latency (s)	Type of cluster	Number of channels incluster
**Theta**			
no-TMS experiment	0–0.25	positive	20
TMS experiment	0.75–1	negative	10
	0–0.25	positive	25
	0.75–1	positive	23
	1–1.25	negative	31
		positive	23
		positive	42
**Alpha**			
no-TMS experiment	0–0.25	positive	17
TMS experiment	0.75–1	negative	33
	1–1.25	positive	9
	0–0.25	negative	22
	0.75–1	positive	11
	1–1.25	negative	14
		positive	8
		negative	25
		positive	14
		negative	27
		positive	15


### Neural Oscillatory Power Before TMS Predicts TMS Response

A linear regression was performed to predict theta power increase after TMS pulse delivery in the time window (1–1.25 s). The results showed a high prediction power from pre-TMS pulse activity (*F*(4,15) = 66.6, *p* < 0.001), explaining up to 93% of the oscillatory activity increase after TMS stimulation. These effects were only significant in the time window 0.75–1 s (*t* = 13, *p* < 0.001). The linear regression showed no significant relationship in alpha power between time windows before (0–1 s) and after (1–1.25 s) TMS pulse delivery.

### BRS Scale Correlations

**FIGURE 8:**
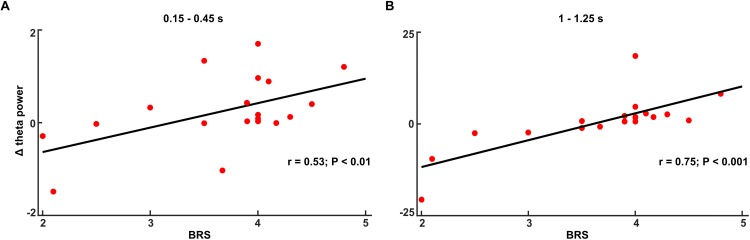
The increased theta power during fear processing (Δ: CS+ – CS–) correlated with Brief Resilience Scale (BRS) score in the time window **(A)** 0.15–0.45 s and **(B)** 1–1.25 s in TMS experiment.

In the no-TMS experiment, the correlation between theta power (0.15–0.45 s) and BRS showed a positive statistical trend (*r* = 0.32, *p* < 0.1). In the TMS experiment, theta power correlated with BRS across all subjects in the time windows (0.15–0.45 and 1–1.25 s) (Figure 8A: 0.15–0.45 s, *n* = 19, *r* = 0.53, *p* < 0.01; Figure 8B: 1–1.25 s, *n* = 19, *r* = 0.75, *p* < 0.001) suggesting that the increases in the theta power during CS+ relative to CS- are related to the individual coping abilities.

## Discussion

In this study, we tracked cortical excitability patterns during fear processing and demonstrated that elevated excitability in the theta generating system of dmPFC could be entrained through single pulse TMS of this region. First, increased excitability of the frontal regions was indicated by increased theta power, but decreased alpha power was found in interconnected occipital regions. These excitability variations were further represented by modulation of functional connectivity in theta and alpha frequency bands. Increased cortical excitability was achieved by delivering single-pulse TMS over the right dmPFC, and the patterns previous to the stimulation clearly determined the modulation of cortical excitability after TMS. The excitability patterns were further associated with a clear behavioral correlate, the individual capability to cope with stressful events, as measured by the BRS.

Previous studies have used subjective fear ratings as an indicative measure of fear induction by the performed task (Fredrikson et al., 1996; Meyer et al., 2015). Further, heart rate acceleration has been proposed as an autonomic index of fear stages (Steimer, 2002). In our study, successful induction of fear in participants in both experiments was evidenced by elevated fear ratings and increased heart rate during CS+ compared to CS-.

### Excitability Patterns During Fear Processing and Their Modulation

Previous studies showed increased cortical excitability to fear stimuli after verbal instruction of the CS+/US contingency (Bublatzky and Schupp, 2012; Weymar et al., 2013; Meyer et al., 2015; Gonzalez-Escamilla et al., 2018a). The current results from GMFP analyses also show increased cortical excitability during processing of fearful stimuli compared to neutral stimuli. The role of theta oscillations has already been described in mice (Likhtik et al., 2014; Karalis et al., 2016) and both theta and alpha oscillations have been described in humans during fear processing (Meyer et al., 2015; Chien et al., 2017; Zheng et al., 2017). In mice, theta oscillations are considered as a mechanism mediating prefrontal-amygdala coupling related to fear expression (Karalis et al., 2016). In humans, increased theta power at specific frontal regions has been suggested as a mechanism of event-related synchronization, whereas decreased alpha power at parietal and occipital sites is related to event-related desynchronization (Chien et al., 2017). In our study, both the experiments evidenced increased theta power across the frontal lobe and decreased alpha power in occipital lobe during fear processing. Our results reproduce the generalizability of robust oscillatory activity in the theta range during fear processing. Furthermore, application of TMS over the right dmPFC induced increased theta and lower alpha power in the same regions in comparison to the pre-stimulation period. These results support the hypothesis that single-pulse TMS modulates spontaneous oscillations emerging from the stimulated region (Rosanova et al., 2009). They therefore provide further evidence for the dmPFC as a key region for the regulation of cortical excitability and appropriate physiological responses to fear processing. We also found evidence for differentiated fear processing in specific temporal windows, highlighting a dynamic process with clearly delimited spatial modulation in both theta and alpha bands.

### Functional Connectivity Patterns During Fear Processing and Their Modulation

The WPLI results showed that in the theta band, the information flow increased from occipital to prefrontal regions, which might represent a key element for the appropriate processing of the threatening event, possibly guiding connectivity patterns among the regions forming the so-called fear network (Chien et al., 2017). In the alpha band, the increased information flow was spatially limited to occipital and parietal regions. The specific short-range connectivity differences in the occipital lobe alpha oscillations could be related to alterations due to the fear processing, which have been shown in earlier studies (Tiitinen et al., 1993; Ponjavic-Conte et al., 2013). After TMS stimulation, the connectivity from sensorimotor regions to prefrontal regions was increased in the theta band, while connectivity in the alpha band occurred in the opposite direction (sensorimotor area to occipital regions). These results suggest a specific balance between excitability (Trenado et al., 2018) and inhibitory mechanisms occurring at different brain areas, as previously suggested (Piantadosi and Floresco, 2014; Lipp et al., 2015), which temporally allow correct processing of fearful stimuli. Such balance is likely to represent a physiological marker for the existence of coping mechanisms, since an impaired excitation and inhibition balance has been largely associated with the development of neuropsychiatric disorders (Selten et al., 2018).

### Predicting the Excitability Modulations

A recent study showed that pre-stimulation measures of cortico-cortical evoked potentials (amplitude, latency, and the distance between stimulation site and channel of interest) predicted modulatory effects following 10 Hz prefrontal repetitive stimulation (Keller et al., 2018). Similarly, our results showed that frontal theta power on the pre-stimulation windows explained the power changes after TMS stimulation (93% of variance explained). This is likely to be of huge future clinical relevance, as it provides evidence for the possibility of accurately identifying target windows that are likely to produce a maximum/optimal modulatory effect on the brain circuits, or could serve as a biomarker in the therapeutic intervention of fear or other mental health disorders. It could therefore be used in future trials for the design of non-invasive treatment procedures.

### Relationship Between Excitability and BRS

We found that people with higher BRS scores showed higher frontal theta power during fear processing. This result suggests that resilient mechanisms play a key role in modulating fear processing. We also showed that people with low BRS score were less susceptible to the modulatory effects of dmPFC-TMS stimulation, suggesting that preserved ability for coping with aversive situations is directly related to specific patterns of cortical excitation and communication within the regions forming fear network. It will be of interest to evaluate this possibility in people suffering with anxiety-related disorders in future studies.

### Limitations

In this study, dmPFC localization was performed with a neuronavigation system based on individual T1-weighted MRI data and coordinates obtained from the previous study (Meyer et al., 2018). Determining the coordinates in each individual by implementation of instructed fear paradigm in fMRI which has shown activation of dmPFC during threat compared to safe (Meyer et al., 2018) might further improve the spatial specificity of the TMS stimulation. In the future studies, it would be interesting to investigate the effect of pre-processing pipeline implemented in this study on functional connectivity measures. Some researchers recommend playing the auditory background noise through headphones to mask the auditory interference caused by the TMS click, and future studies could consider this approach.

## Conclusion

In summary, our results provide insight into the dynamics of cortical excitability modulation and functional connectivity during fear processing, while the patterns of pre-stimulation frontal theta power determine the magnitude of effects induced by TMS stimulation. Moreover, frontal theta power is clearly related to an individual’s ability to cope with challenging situations, leaving those individuals with low coping abilities more vulnerable to functional failure in face of adversity.

## Author Contributions

VC contributed to data acquisition, data analysis, and manuscript writing. NK, TB, and SvG contributed to data acquisition. GG-E contributed to data discussion and manuscript writing. MM and SG contributed to experimental design, data analysis, and revision of the manuscript.

## Conflict of Interest Statement

The authors declare that the research was conducted in the absence of any commercial or financial relationships that could be construed as a potential conflict of interest.

## Footnotes

1 http://www.vislab.ucl.ac.uk/cogent_2000.php

2 http://www.fieldtriptoolbox.org

3 http://www.statistica.com
