# Supplementary figures and images for: Cortical Excitability Dynamics During Fear Processing
Source: Front Neurosci. 2019 Jun 4;13:568. doi: 10.3389/fnins.2019.00568 (PMC6593288; doi:10.3389/fnins.2019.00568)

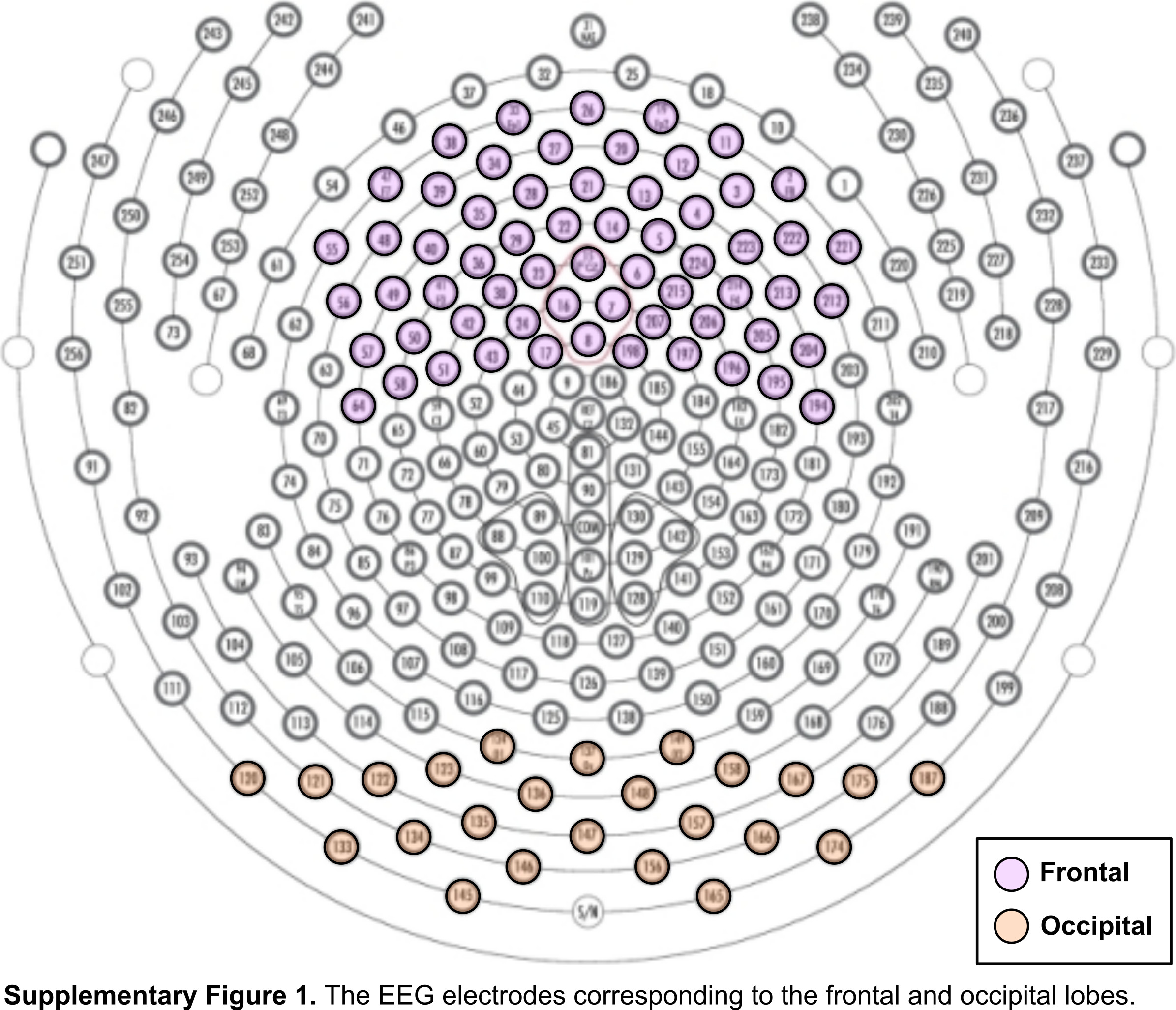

Supplement: Supplementary file 1 [file Image_1.jpeg]
